# Intestinal Fatty Acid-Binding Protein as a Diagnostic Marker for Complicated and Uncomplicated Necrotizing Enterocolitis: A Prospective Cohort Study

**DOI:** 10.1371/journal.pone.0121336

**Published:** 2015-03-20

**Authors:** Maarten Schurink, Elisabeth M. W. Kooi, Christian V. Hulzebos, Rozemarijn G. Kox, Henk Groen, Erik Heineman, Arend F. Bos, Jan B. F. Hulscher

**Affiliations:** 1 Department of Paediatric Surgery, University of Groningen, University Medical Centre Groningen, Groningen, the Netherlands; 2 Department of Neonatology, University of Groningen, Beatrix Children's Hospital, University Medical Centre Groningen, Groningen, the Netherlands; 3 Surgical Research Laboratory, University of Groningen, University Medical Centre Groningen, Groningen, the Netherlands; 4 Department of Epidemiology, University of Groningen, University Medical Centre Groningen, Groningen, the Netherlands; Indian Institute of Science, INDIA

## Abstract

**Background:**

Early NEC symptoms are non-specific and diagnostic tests lack discriminative power. Intestinal fatty acid-binding protein (I-FABP), mainly located in small bowel enterocytes, is released into the blood following NEC-associated enterocyte disruption. Aim of this prospective cohort trial was to determine the diagnostic value of I-FABP measured in plasma (I-FABPp) and urine (I-FABPu) for the presence of NEC, to evaluate I-FABP levels during NEC development, and to assess its prognostic value for the progression from suspected to complicated disease.

**Methods:**

Between 2010 and 2012 we prospectively enrolled neonates with suspected NEC. We measured I-FABP levels eight-hourly from onset of suspected NEC for at least 48 hours, or until surgery. NEC diagnosis was confirmed radiologically or during operation. We defined NEC as complicated if it resulted in surgery and/or death. We determined disease course and diagnostic I-FABP cut-off points.

**Results:**

The study comprised 37 neonates (24M, 13F), gestational age 28 (24–36) weeks, birth weight 1190 (570–2,400) grams. We found significantly higher I-FABPp and I-FABPu levels in NEC patients (n = 22) than in patients with other diagnoses (n = 15). Cut-off values for diagnosing NEC were 9 ng/mL I-FABPp and 218 ng/mL I-FABPu, with corresponding likelihood ratios (LRs) of 5.6 (95% CI 0.89–35) and 5.1 (95% CI 0.73–36), respectively. I-FABP levels were highest in the first eight hours after symptom onset and gradually decreased over time. Cut-off values for complicated disease were 19 ng/mL I-FABPp and 232 ng/mL I-FABPu, with LRs of 10 (95% CI 1.6–70) and 11 (95% CI 1.6–81), respectively.

**Conclusions:**

Both plasma and urinary I-FABP levels specifically identify NEC in preterm infants prior to appearance of diagnostic radiological signs suggestive for NEC. Moreover, serial I-FABP measurements accurately predict development of complicated disease.

## Introduction

Early symptoms of necrotizing enterocolitis (NEC) are often non-specific, such as abdominal distension, bloody stools, or gastric retention [1,2]. Early identification of those patients who will eventually develop definite NEC remains challenging, primarily because current laboratory and radiology tests lack sufficient discriminative power [3,4].

Because NEC is characterized by loss of bowel wall integrity, intestinal fatty acid-binding protein (I-FABP) is one of the more promising biomarkers. This small cytosolic protein, located mainly in enterocytes of the small intestine, is released into the blood stream after cell disruption [5–7]. I-FABP is readily excreted by the kidneys and can, therefore, be measured both in plasma and in urine within hours of tissue damage [8,9].

While several studies investigated the discriminative power of I-FABP for NEC at onset of disease, no study evaluated changes in I-FABP levels during its development. As NEC is often a progressive disease, consecutive measurements might offer more detailed information about the disease course than a single measurement at first symptoms.

Our aim was threefold: to determine the diagnostic value of plasma and urine I-FABP for the presence of definite NEC, to evaluate plasma and urine I-FABP levels during NEC development, and to assess the prognostic value of plasma and urine I-FABP for the progression from suspected to complicated disease (as defined by disease resulting in a surgical intervention and/or death).

## Methods

The study has been approved by the Institutional Review Board of the University Medical Centre Groningen, the Netherlands, and has been conducted according to the principles expressed in the Declaration of Helsinki. Written informed consent was obtained from (the parents of) all participants.

As part of the prospective cohort trial ‘NoNEC’ (number NTR3239 in the Dutch Trial Registry), all consecutive patients with suspected NEC, admitted to the neonatal intensive care unit (NICU) of University Medical Centre Groningen between October 2010 en October 2012, were included [10]. Suspected NEC was defined as Bell’s stage I, in which only non-specific symptoms, such as gastric retention, abdominal distension, or mild ileus are present [1,2].

All data were prospectively collected and included demographics (gender, gestational age, and birth weight), other patient data (type of delivery, medication, and co-morbidity), maternal data, and disease characteristics.

On first presentation of symptoms, all patients were treated according to hospital protocol: bowel rest (i.e. nil by mouth and decompression by nasogastric suction), broad-spectrum antibiotics (after cultures were obtained), pain management, and respiratory and/or cardiovascular support if necessary. Regular diagnostic laboratory and radiological studies, such as an abdominal X-ray, were performed. The latter was regarded as onset of symptoms, because the time of the abdominal X-ray was well documented in the digital hospital information system and it was performed immediately after suspicion of NEC arose. Specifying the exact time of symptom onset was considered essential information for the purpose of delineating the course of disease. If a patient was transferred to our centre because of suspected NEC, time of abdominal X-ray in the referring hospital was considered as time of symptom onset. Following as soon as possible the first study samples of blood and urine were obtained.

Diagnosis of NEC was defined as the presence of pneumatosis intestinalis on abdominal X-ray (and/or peroperatively confirmed) and corresponding to Bell’s stage II or higher [1,2]. Complicated disease was defined as a clinical condition resulting in a surgical intervention and/or death. Indications for surgical intervention were clinical deterioration despite maximal conservative therapy or signs of pneumoperitoneum. In NEC patients, complicated disease often corresponds to an advanced Bell’s stage including evidence for bowel perforation, i.e. Bell’s stage III^B^. An expert panel of consultant neonatologists and paediatric surgeons, blinded as to the I-FABP levels, determined the final Bell’s stage of each patient. Patients who did not develop definite NEC were considered no-NEC patients.

Together with every routine diagnostic blood analysis an extra sample of 100 μL was obtained in an EDTA tube for study purposes. Urine samples were collected at regular intervals by placing cotton in the patient’s nappy. Once it was saturated with urine, the urine was gently squeezed into a sterile syringe and then pressed in a standard urine tube. In patients with an indwelling catheter, urine was collected directly from the catheter. Blood and urine samples were put on ice and sent to the laboratory without delay. Ideally, both blood and urine samples were collected every eight hours. If, however, the clinical condition of the patient dictated otherwise, only a urine sample was obtained, thus minimizing the burden for the patient.

Sampling blood and urine for study purposes ended either if ten consecutive samples of blood and/or urine had been obtained during at least 48 hours, if the patient underwent a surgical intervention (because such an intervention would of itself probably cause a non-physiologic rise in I-FABP levels), or if the patient died.

In the laboratory, a blood sample was fractioned by centrifuging it for 10 minutes at approximately 2,000 x *g*. Plasma was then aspirated and put in a 0.5 mL Sarstedt tube and stored at -80°C. Approximately 1.5 mL of urine was transferred from the urine tube to a 2 mL Sarstedt tube and also stored at -80°C. After study participation of all patients was completed, a laboratory technician, who was blinded as to the patient characteristics, performed plasma and urinary I-FABP measurements. Commercially available ELISAs were used for both urine and plasma I-FABP measurements (Human FABP2 kit from R&D systems, Minneapolis, MN, USA).

### Statistical analysis

Because this study was essentially exploratory in nature, we refrained from a power analysis.

Statistical analysis was performed using the Statistical Package for the Social Sciences (IBM SPSS Statistics 21, IBM Corp., Armonk, New York, USA). All data are presented as median with (range), unless specified otherwise. Two-tailed *P* values of less than 0.05 were considered statistically significant. For categorical variables the chi-square test was used and for continuous variables the *t* test or Mann-Whitney test, as appropriate. To determine statistical difference between patient groups (i.e. no-NEC patients and patients with complicated or uncomplicated NEC, respectively) 2-way Mann-Whitney tests were performed. For the purpose of constructing graphs, data were logarithmically transformed if necessary (and a constant ‘1’ was added to avoid negative numbers, if applicable).

To determine the clinical use of plasma and urine I-FABP levels as a diagnostic test for suspected NEC, positive and negative likelihood ratios were computed after constructing receiver operating characteristic curves. Cut-off points were determined on the basis of the optimum sum of sensitivity and specificity. To avoid erroneously establishing the diagnosis of NEC or unnecessary operative interventions based on false positive I-FABP values, a high specificity was preferred. To determine the clinical use of plasma and urine I-FABP levels as a prognostic test for complicated disease, similar methods were applied.

For the purpose of delineating the course of disease, I-FABP measurements were classified in six time slots, i.e. 0–8 h, 8–16 h, 16–24 h, 24–36 h, 36–48 h, and >48 h after symptom onset, based on the number of hours that had elapsed since symptom onset (identified by the time of first abdominal X-ray). Samples were classified according to these time slots.

## Results

**Fig 1:**
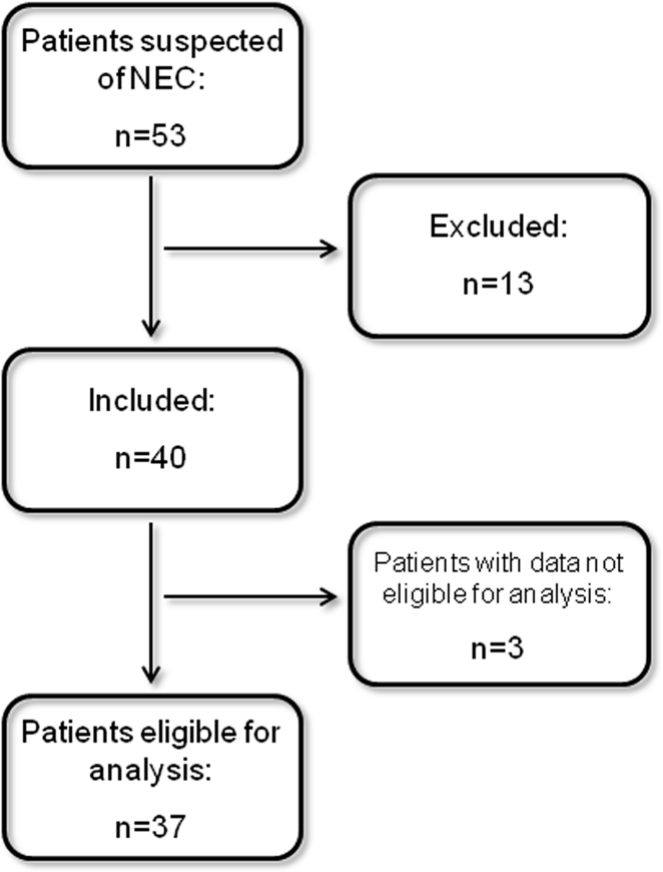
Flow chart of patient inclusion.

During the study period 53 consecutive patients with suspected NEC were admitted to the NICU and eligible for inclusion. Thirteen patients could not be included: 12 because informed consent was withheld and one because of the already terminal nature of the prognosis on presentation (Fig. 1). There were no statistically significant differences between the included and excluded patients except for gestational age: the excluded patients were younger (median 26 versus 28 weeks, *P* = 0.04). The remaining 40 patients were included in the study, but the laboratory data of three could not be analyzed for logistic reasons.

In Table 1 we summarize the demographic data of the 37 patients suitable for analysis, 24 boys and 13 girls with a median gestational age of 28 weeks (24–36) and median birth weight of 1190 grams (570–2400]. They developed Bell’s stage I symptoms at a median postnatal age of 9 days (3–43), upon which the first diagnostic abdominal X-ray was obtained. After a median of 7.5 hours (1–44) after symptom onset, study participation and collecting of blood and urine samples commenced. Five patients were referred to our tertiary care centre with suspected NEC. This diagnosis was confirmed by additional abdominal X-rays in all cases. In 22 out of 37 patients (59%) suspected NEC progressed to definite NEC: in 15/22 (68%) NEC patients, diagnosis was confirmed by the abdominal X-ray performed at onset of symptoms. In the other seven cases NEC was confirmed after 0–8 h (n = 2), 8–16 h (n = 1), 16–24 h (n = 1) or 24–48 h (n = 3). Final Bell’s stages of these 22 infants and diagnoses of the 15 no-NEC patients are summarized in Table 1.

**Table 1:** Characteristics of included patients.

	N = 37
Gestational age	28 wk (24–36)
Birth weight	1,190 g (570–2,400)
Gender: male/female	24/13
Postnatal age at first symptoms	9 d (3–34)
Diagnosis of NEC:	22/37 (59%)
Final Bell’s stage II^A^	9/22 (41%)
II^B^	2/22 (9%)
III^A^	2/22 (9%)
III^B^	9/22 (41%)
NICU stay	27 d (8–102)

Values are expressed as median (range). NICU = neonatal intensive care unit.

The remaining 15 patients were diagnosed with ileus caused by sepsis e.c.i. (e causa ignota; n = 3), delayed passage of meconium (n = 2), bloody stool e.c.i. (n = 2), CPAP belly (n = 2), (viral) gastroenteritis (n = 2), spontaneous intestinal perforation (SIP; n = 1), and sigmoid volvulus (n = 1). In two patients no definite diagnosis could be made.

### Diagnostic value of I-FABP for the presence of NEC

**Fig 2:**
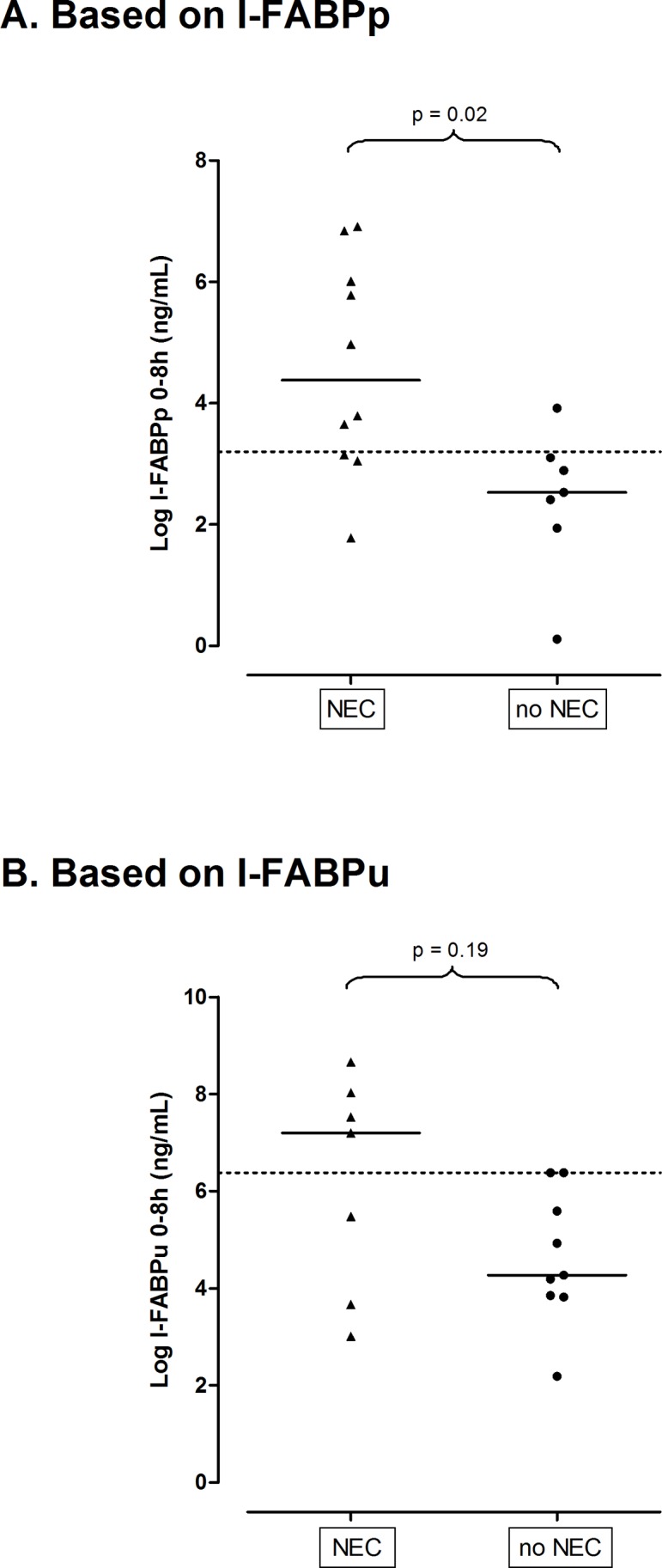
Median I-FABP values (after logarithmic transformation), measured from 0–8 h in plasma (A) and urine (B), of 22 NEC versus 15 no-NEC patients. Cut-off points to differentiate between groups are represented by dotted lines (after logarithmic transformation: cut-off points at 3.2 and 6.4 ng/mL correspond to cut-off points at 9 and 218 ng/mL, as mentioned in tables 2A and 2B, respectively). A. Based on I-FABPp. B. Based on I-FABPu.

Between 0–8 h after symptom onset, I-FABPp (17 samples) and I-FABPu (16 samples) values were 35 (2.2–370) and 491 (7.5–2 116) ng/mL in NEC patients (n = 22), versus 4.6 (0.41–19) and 26 (3.3–218) ng/mL in patients without NEC. Only I-FABPp values reached statistical significance when both groups were compared (*P* = 0.02 and *P* = 0.19, respectively). In Fig. 2 (A and B), we present I-FABP values for diagnosis of NEC after logarithmic transformation. We calculated optimal cut-off points to distinguish NEC from no-NEC patients (Table 2, A and B). Using cut-off values of 9 ng/mL I-FABPp and 218 ng/mL I-FABPu, positive LRs for the diagnosis of NEC within eight hours after symptom onset were 5.6 (0.89–35) and 5.1 (0.73–36), respectively. For each time slot following the first eight hours we calculated optimal cut-off values, while aiming at a high specificity. LRs remained sufficiently high throughout the next 40 hours to confirm the diagnosis of NEC. Highest LRs were 5.3 (0.78–36) and 6.6 (1.0–43) for I-FABPp and I-FABPu, observed at 24–36 h and 16–24 h respectively (Table 2, A and B). If abovementioned 0–8 h cut-off values of 9 ng/mL I-FABPp and 218 ng/mL I-FABPu were applied to all following time slots, sensitivities for both I-FABPp and I-FABPu dropped to 11–77% and 5–50% respectively, with specificities soon equalling 100% (data not shown).

**Table 2:** Cut-off Values, likelihood ratios, and other test characteristics of I-FABPp (A) and I-FABPu (B) as a predictive test for the presence of NEC from 0–48 h after onset of symptoms (n = 37).

A. Based on I-FABPp							
h	n	Median values (range)	Cut-off values (ng/mL)	Sensitivity (%)	Specificity (%)	LR+ (95% CI)	LR- (95% CI)
0–8	17	8.6 (0.41–370)	9	80	86	5.6 (0.89–35)	0.23 (0.06–0.84)
8–16	20	11 (0.44–3748)	11	69	86	4.8 (0.76–31)	0.36 (0.15–0.85)
16–24	19	7.3 (1.1–809)	10	64	88	5.1 (0.77–34)	0.42 (0.18–0.94)
24–36	27	3.4 (0.25–113)	7	53	90	5.3 (0.78–36)	0.52 (0.31–0.88)
36–48	25	2.0 (0.79–35)	2	67	86	4.7 (0.74–29)	0.39 (0.19–0.78)
>48	30	2.8 (1.1–141)	3	67	75	2.7 (0.95–7.5)	0.44 (0.22–0.90)
I-FABPp = intestinal fatty acid-binding protein in plasma; LR+ = positive likelihood ratio; LR- = negative likelihood ratio; CI = confidence interval.							

B. Based on I-FABPu							
h	n	Median values (range)	Cut-off values (ng/mL)	Sensitivity (%)	Specificity (%)	LR+ (95% CI)	LR- (95% CI)
0–8	16	69 (3.3–2116)	218	57	89	5.1 (0.73–36)	0.48 (0.20–1.2)
8–16	23	36 (3.2–23,337)	90	58	91	6.4 (0.93–44)	0.46 (0.23–0.91)
16–24	24	51 (1.9–18,267)	52	73	89	6.6 (1.0–43)	0.30 (0.13–0.71)
24–36	32	26 (1.3–1 620)	85	28	93	3.9 (0.51–30)	0.78 (0.58–1.1)
36–48	30	16 (1.8–857)	34	53	91	5.8 (0.85–39)	0.52 (0.32–0.85)
>48	34	17 (0.56–3596)	96	21	93	3.2 (0.39–25)	0.85 (0.66–1.1)
I-FABPu = intestinal fatty acid-binding protein in urine; LR+ = positive likelihood ratio; LR- = negative likelihood ratio; CI = confidence interval.							

### I-FABP levels during NEC development

**Fig 3:**
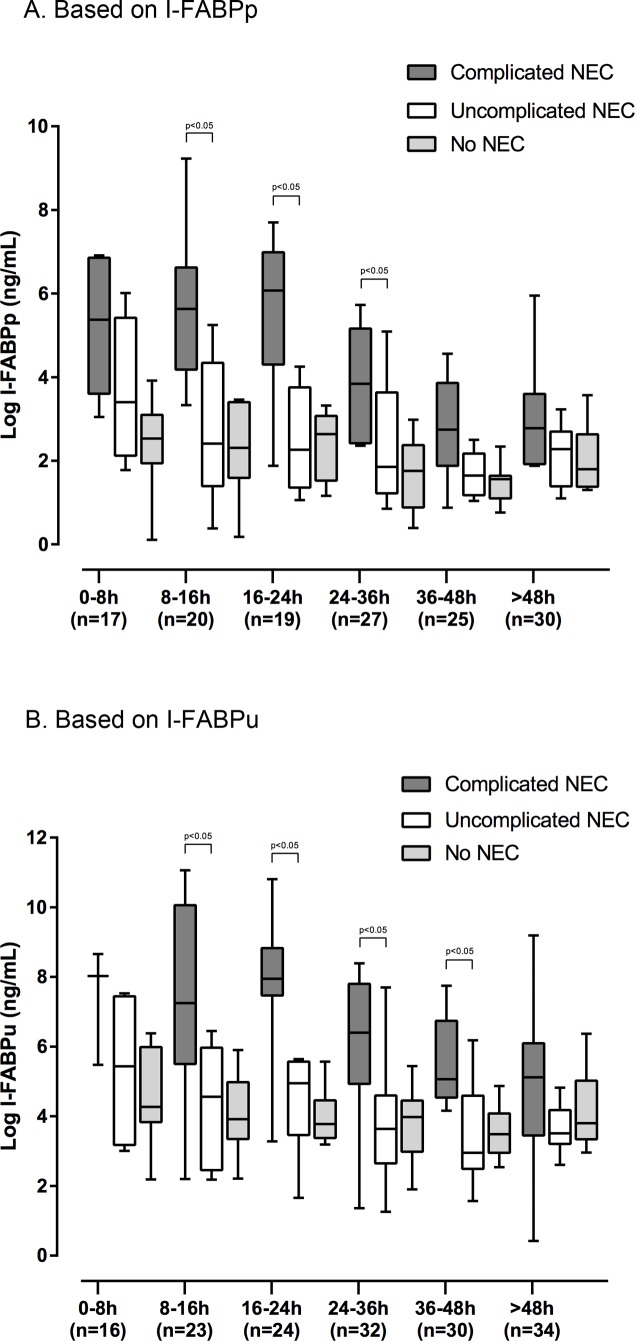
Box plots depicting median I-FABP values (after logarithmic transformation) measured in plasma (A) and urine (B) of all patients during the course of disease. A. Based on I-FABPp. B. Based on I-FABPu.

I-FABPp and I-FABPu levels were highest in the first 24 hours after symptom onset (as defined by the first abdominal X-ray) and tended to decrease gradually over time (Fig. 3, A and B). I-FABPp levels in NEC patients remained higher than those in no-NEC patients during the first 36 hours. As median I-FABP levels tended to decrease over time, so did the cut-off values distinguishing between NEC and no-NEC (Table 2, A and B).

Fig. 3 (A and B) shows I-FABP levels during the disease course of patients with complicated and uncomplicated NEC plotted against patients without NEC. Only the differences between patients with and without complicated NEC were statistically significant in several time slots and are marked accordingly in the two figures.

The specificity for I-FABP to determine whether patients would develop complicated NEC gradually increased over time, ranging from 75% at onset of disease to 91% after 48 hours (Table 3, A and B).

**Table 3:** Cut-off values, likelihood ratios, and other test characteristics of I-FABPp (A) and I-FABPu (B) as a predictive test for the development complicated NEC (after NEC has been diagnosed) from 0–48 h after onset of symptoms (n = 22).

A. Based on I-FABPp							
h	n	Median values (range)	Cut-off values (ng/mL)	Sensitivity (%)	Specificity (%)	LR+ (95% CI)	LR- (95% CI)
0–8	10	35 (2.2–370)	53	67	75	2.7 (0.45–16)	0.44 (0.12–1.6)
8–16	13	53 (0.54–3748)	19	88	80	4.4 (0.74–26)	0.16 (0.02–1.1)
16–24	11	27 (1.1–809)	26	86	75	3.4 (0.61–19)	0.19 (0.03–1.4)
24–36	16	11 (0.86–113)	29	43	90	4.3 (0.55–33)	0.63 (0.33–1.2)
36–48	18	2.9 (0.89–1620)	4	63	90	6.3 (0.90–43)	0.42 (0.17–1.0)
>48	18	3.6 (1.1–141)	9	43	91	4.7 (0.60–37)	0.63 (0.33–1.2)
I-FABPp = intestinal fatty acid-binding protein in plasma; LR+ = positive likelihood ratio; LR- = negative likelihood ratio; CI = confidence interval.							

B. Based on I-FABPu							
h	n	Median values (range)	Cut-off values (ng/mL)	Sensitivity (%)	Specificity (%)	LR+ (95% CI)	LR- (95% CI)
0–8	7	491 (7.5–2116)	687	67	75	2.7 (0.41–17)	0.44 (0.08–2.6)
8–16	12	161 (3.2–23,337)	232	71	80	3.6 (0.58–22)	0.36 (0.10–1.3)
16–24	15	103 (1.9–18,267)	103	88	86	6.1 (0.98–38)	0.15 (0.02–0.9)
24–36	18	32 (1.3–1620)	222	57	91	6.3 (0.87–45)	0.47 (0.20–1.1)
36–48	19	34 (1.8–857)	177	38	91	4.1 (0.52–33)	0.69 (0.39–1.2)
>48	19	19 (0.56–3596)	46	90	91	5.5 (0.75–40)	0.55 (0.27–1.1)
I-FABPu = intestinal fatty acid-binding protein in urine; LR+ = positive likelihood ratio; LR- = negative likelihood ratio; CI = confidence interval.							

### Prognostic value of I-FABP for the progression to complicated disease

Within eight hours after symptom onset, I-FABPp and I-FABPu values were higher in patients with complicated disease (n = 12: 11 infants with complicated NEC and one infant with a spontaneous intestinal perforation, SIP) than in patients without complicated disease, although statistical significance was not unequivocal for both I-FABPp and I-FABPu (*P* = 0.02 and *P* = 0.05, respectively).

Between 8–16 h after symptom onset, I-FABPp and I-FABPu values were 103 (10–3,748) and 517 (3.2–23,337) ng/mL in patients with complicated disease versus 4.0 (0.44–70) and 22 (3.2–232) ng/mL in patients without complicated disease with associated *P* values of 0.001 and 0.009, respectively. In daily practice, the first hours after symptom onset will be used to confirm the diagnosis. Once a diagnosis is confirmed, information regarding the expected severity and course of the disease becomes more important. Therefore, we chose to calculate the optimal cut-off points to distinguish between patients with and without complicated disease for time slot 8–16 h (Table 4, A and B). Optimal cut-off values of 19 ng/mL I-FABPp and 232 ng/mL I-FABPu were calculated, with positive LRs for predicting the development of complicated disease of 10 (1.6–70) and 11 (1.6–81), respectively. After these first 16 hours, positive LRs remained high with a gradual increase in specificity of more than 94%. When these calculations were limited to patients with complicated NEC (n = 11), the same cut-off values applied, albeit with less pronounced LRs (Table 3, A and B). If 0–8h cut-off values of 53 ng/mL I-FABPp and 687 ng/mL I-FABPu were applied to all following time slots, sensitivities for both I-FABPp and I-FABPu ranged from 0–75% and 11–50% respectively, with specificities soon equalling 100% (data not shown).

**Table 4:** Cut-off values, likelihood ratios, and other test characteristics of I-FABPp (A) and I-FABPu (B) as a predictive test for the development of complicated disease (both NEC and otherwise) from 0–48 h after onset of symptoms (n = 37).

A. Based on I-FABPp							
h	n	Median values (range)	Cut-off values (ng/mL)	Sensitivity (%)	Specificity (%)	LR+ (95% CI)	LR- (95% CI)
0–8	17	8.6 (0.41–370)	53	67	91	7.3 (1.0–52)	0.37 (0.12–1.2)
8–16	20	11 (0.44–3748)	19	88	92	10 (1.6–70)	0.14 (0.02–0.87)
16–24	19	7.3 (1.1–809)	26	86	92	10 (1.5–69)	0.16 (0.03–0.97)
24–36	27	3.4 (0.25–113)	29	43	95	8.6 (1.1–70)	0.60 (0.32–1.2)
36–48	25	2.0 (0.79–35)	4	56	94	8.9 (1.2–65)	0.47 (0.23–0.99)
>48	30	2.8 (1.1–141)	10	50	95	11 (1.4–84)	0.52 (0.26–1.1)
I-FABPp = intestinal fatty acid-binding protein in plasma; LR+ = positive likelihood ratio; LR- = negative likelihood ratio; CI = confidence interval.							

B. Based on I-FABPu							
h	n	Median values (range)	Cut-off values (ng/mL)	Sensitivity (%)	Specificity (%)	LR+ (95% CI)	LR- (95% CI)
0–8	16	69 (3.3–2116)	687	67	92	8.7 (1.1–67)	0.36 (0.07–1.8)
8–16	23	36 (3.2–23,337)	232	71	94	11 (1.6–81)	0.30 (0.09–0.99)
16–24	24	51 (1.9–18,267)	103	88	94	14 (2.1–95)	0.13 (0.02–0.84)
24–36	32	26 (1.3–1620)	222	57	96	14 (1.9–108)	0.47 (0.19–1.1)
36–48	30	16 (1.8–857)	177	38	95	8.3 (1.0–68)	0.65 (0.38–1.1)
>48	34	17 (0.6–3 596)	96	44	96	11 (1.4–87)	0.58 (0.32–1.0)
I-FABPu = intestinal fatty acid-binding protein in urine; LR+ = positive likelihood ratio; LR- = negative likelihood ratio; CI = confidence interval.							

**Fig 4:**
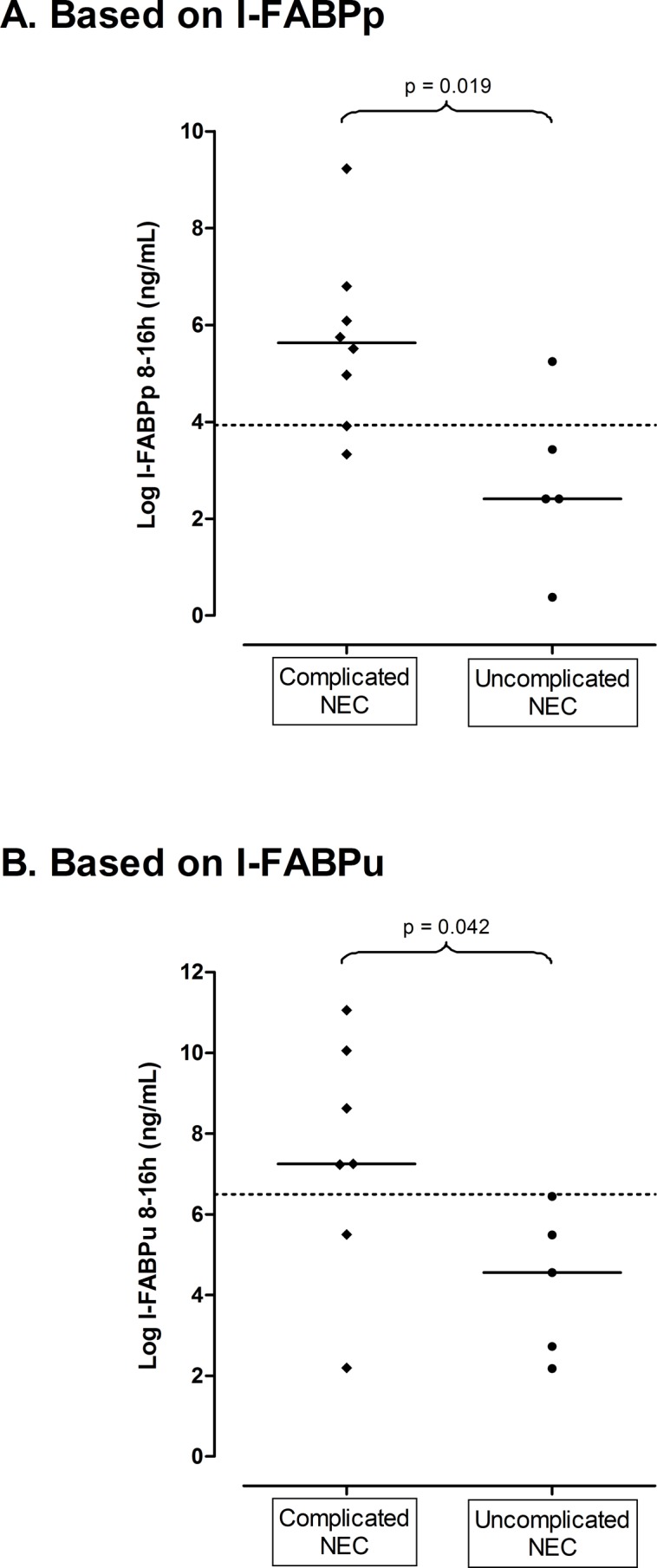
Median I-FABP values (after logarithmic transformation), measured in plasma (A) and urine (B) from 8–16 h, of patients with complicated NEC (n = 11) versus uncomplicated NEC (n = 11). Cut-off points to differentiate between groups are represented by dotted lines (after logarithmic transformation: cut-off points at 3.9 and 6.5 ng/mL correspond to cut-off points at 19 and 232 ng/mL, as mentioned in tables 4A and 4B, respectively). A. Based on I-FABPp. B. Based on I-FABPu.

When the analysis is being limited to (all) NEC patients, 11 patients with complicated NEC versus 11 patients with uncomplicated NEC showed statistically significant different I-FABP levels, whether measured in plasma (103 [10–3,748] ng/mL versus 4.1 [0.54–70] ng/mL; p = 0.019) or urine (517 [3.3–23,337] ng/mL versus 35 [3.2–232] ng/mL; p = 0.042) at 8–16 h after onset of disease (Fig. 4, A and B).

### Therapy and outcome

Ten out of 37 patients (27%) underwent a surgical intervention (i.e. laparotomy; no peritoneal drainage was performed in this patient cohort). Nine (90%) patients were operated because of NEC. This intervention occurred median 55 hours (3–823) after symptom onset. The patient who underwent a laparotomy after 823 hours was initially conservatively treated but turned out to have suffered a NEC totalis. He died shortly after the operation. None of the patients were unable to undergo surgical intervention, e.g. because of contra-indications, and subsequently succumbed. Three patients underwent surgery because of free intra-peritoneal air and six patients because of clinical deterioration despite maximal conservative therapy. In four of the latter patients a covered perforation was found during surgery. Median I-FABP levels in plasma and urine were not statistically different between patients with and without a radiologically proven perforation. The tenth patient was operated because of SIP 177 hours after symptom onset. The initial plasma and urine I-FABP levels of this particular patient were 0.79 ng/mL and 4.5 ng/mL, respectively, and highest preoperative I-FABP levels were 13 ng/mL and 63 ng/mL, respectively.

**Fig 5:**
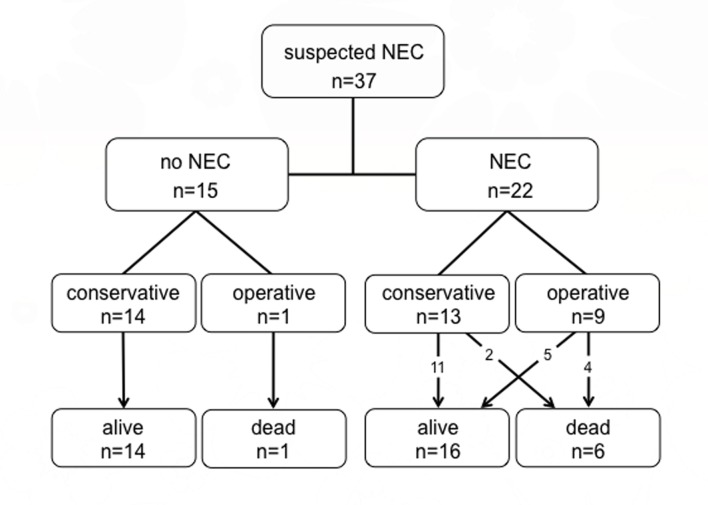
Flow chart of patient outcome.

Patient outcome is shown in Fig. 5. Overall mortality was 7 out of 37 (19%): 6 succumbed to the sequelae of NEC. The seventh patient was the patient operated on because of SIP.

## Discussion

### Diagnostic value of I-FABP at onset of symptoms

Our study shows that in neonates classified as Bell’s stage I, I-FABP measured in plasma at symptom onset can identify those patients in whom non-specific symptoms will evolve into definite NEC. A cut-off point for I-FABPp was determined at 9 ng/mL, associated with a positive LR of 5.6. The corresponding high specificity minimizes the chance of erroneously establishing the diagnosis of NEC.

Several earlier studies reported on I-FABP measured in plasma and serum [5, 11–15]. In their first quantitative radioimmunoassay for human I-FABP, Liebermann et al. reported that I-FABPp might serve as a diagnostic marker for early intestinal mucosal compromise, such as NEC [5]. Our results confirmed their findings; additionally we provide data on the infants’ Bell’s stages and the time course of I-FABP levels. Several studies found I-FABPp to be a specific marker for early identification of severe NEC (Bell’s stage III), but less useful for differentiating Bell’s stages I and II [11,12]. This was also the case in our series: the difference between the NEC and no-NEC patients was due mainly to NEC cases that eventually progressed towards complicated NEC. In our study, patients with suspected NEC, who eventually were not diagnosed with NEC, could be considered controls, thereby reflecting everyday practice. In 2011, Thuijls et al. concluded, on the basis of single samples, that I-FABPp could improve early diagnosis of intestinal ischemia [13]. In the same year, a study on I-FABP measured in serum instead of plasma, reported on a cut-off value for the diagnosis of NEC, but only if symptoms had been present for at least 72 hours [15]. Again, our results demonstrated that as early as the first eight hours after onset of non-specific gastro-intestinal symptoms, I-FABPp had valuable diagnostic and prognostic properties. In 2013, a case-controlled study by Ng and colleagues showed that I-FABPp is a potentially useful biomarker for differentiating between NEC and septic patients and/or control patients with suspected clinical sepsis or NEC [14]. Similar to our results, they found significantly higher I-FABPp levels in surgical NEC infants than in non-surgical infants. More recently, in a study comparing I-FABP with other potential markers, Benkoe et al reported too that I-FABP concentrations were significantly higher in patients in NEC infants compared with controls [16].

Urine analysis represents a good alternative for I-FABPp measurements because every blood sample taken means an extra burden for the neonate [17,18]. When early diagnosis of NEC was based on I-FABP measured in urine, our results clearly showed that a cut-off value of 218 ng/mL had an associated LR of 5.1 (0.73–36) with a correspondingly high specificity. Measuring I-FABPu has several additional advantages over I-FABPp. I-FABP molecules present in plasma will pass the glomerular filter (fractional excretion 28%; half time 11 minutes) without significant degradation of their immunogenic epitopes and are easily detected in urine [5,8,9]. Because the bladder serves as a storage site, it allows for identification of I-FABP over a longer period of time, even after detectable levels have been cleared from the blood. This is consistent with our finding of higher I-FABP levels in urine than in plasma. Several papers reported on the use of urinary I-FABP as a biomarker for intestinal ischemia, NEC, or suspected NEC [5,9,13,19–21]. Our results showed that I-FABPu was not only a useful biomarker for the presence of NEC, but could also, in the earliest stages of disease, distinguish between those infants who developed complicated NEC and infants who could be treated conservatively and successfully.

Although no interventions other than the aforementioned conservative measures (i.e. gastric decompression and broad-spectrum antibiotics) exist to prevent the progression of NEC, I-FABP, whether measured in plasma or urine, provides the clinician with a valuable tool: if patients, who eventually do not develop NEC, can be identified at the time when only non-specific symptoms are present, NEC-specific treatment can be avoided and duration of conservative treatment can be limited. Re-introducing enteral feeding and early discontinuation of antibiotic treatment would be among the main benefits. Alternatively, if progression to NEC is suspected, necessary measures, such as an earlier surgical intervention, could be warranted. As already mentioned, cut-off values for I-FABP determined in our study correspond to a high specificity to minimize the chance of erroneously establishing the diagnosis of NEC. By performing serial I-FABP measurements over a short course of time (e.g. every 4–8 hours) the chance of false negative results will be further minimized. If the cut-off values calculated in the first time slot (i.e. 0–8 h after onset of symptoms) would have been applied to all following time slots, results showed very low sensitivity values in combination with specificity values soon equalling 100%, thereby rendering calculations of positive likelihood ratios impossible. Cut-off values for each time slot were therefore calculated.

### I-FABP levels during NEC development

To the best of our knowledge, our study is the first to demonstrate I-FABP levels in plasma and urine during the course of NEC development. One of the most striking characteristics of I-FABP levels plotted against time is the peak early-on in the course of disease, i.e. within 24 hours after symptom onset. From that moment on, the gradual decline in I-FABP levels is only interrupted by minor fluctuations. A possible explanation for this course in I-FABP levels starts with the fact that I-FABP is expressed in mature enterocytes (rather than in the crypts) and that these cells are located at the most distal point from mucosal blood supply [5]. Subsequently, when the gut is compromised by ischemia and enterocytes are damaged as a consequence, I-FABP is released into the blood stream. This most likely explains the peak in I-FABP levels in the earliest phases of disease. Because intestinal compromise is still reversible at this stage—which explains the success of conservative management of mild NEC cases—I-FABP levels probably correlate well with the extent of the disease [5,11,12, 19]. If one takes into account the fact that I-FABP is located in the tip of the villi, disease extension is most likely specifically correlated to longitudinal extension, rather than progression to transmural involvement. One could expect I-FABP to remain measurable given continued extension of bowel involvement [19]. This seems to be in line with our finding that the only SIP patient in our series had relatively low I-FABP levels. With progression to more severe disease, I-FABP levels diminish until the kidneys have cleared all I-FABP, as mucosal stores of I-FABP are finite. In case of transmural intestinal necrosis or if blood supply to a segment of bowel is irretrievably lost due to demarcation, no more I-FABP will be released into the blood stream. This probably means that a return to normal, or near-normal, I-FABP levels cannot be interpreted solely as an indication that surgical intervention is not warranted, but alternatively that the infant’s condition is ameliorating. In the present series the highest I-FABP levels were found in patients with complicated disease, several of whom underwent a surgical intervention. Because such an intervention of itself probably causes a non-physiologic rise in I-FABP levels, sampling of I-FABP was ceased upon operation. This probably explains why cut-off values distinguishing between patients with and without NEC decreased over time as the patients with highest I-FABP levels were removed from the NEC cohort due to surgical intervention. The specificity of predicting complicated disease gradually increased over time and reached its maximum in the >48 h time slot. This suggests that sequential I-FABP measurements will provide an even more accurate tool to facilitate clinical decision-making.

### Prognostic value of I-FABP for the progression to complicated disease

Within 8–16 hours after symptom onset, I-FABP, whether measured in plasma or urine, could predict the development of complicated disease with cut-off values of 19 ng/mL I-FABPp and 232 ng/mL I-FABPu, with associated positive LRs of 10 (1.6–70) and 11 (1.6–81), respectively. By classifying patients according to the previously mentioned method, even patients without NEC, but with an indication for laparotomy (e.g. SIP), are being acknowledged. This also holds true when we focus on NEC patients. Patients who develop complicated NEC can also be identified early during the course of disease. It is tempting to speculate that measuring I-FABP levels might help to identify patients undergoing early surgery, thus possibly preventing further clinical deterioration. A specific example from our study cohort clearly stresses the importance of previously mentioned speculation: I-FABP levels of the patient with NEC totalis, who was operated on (after 823 hours) following a protracted conservative treatment (see also paragraph ‘Therapy and outcome’ of the Results), were 91 ng/mL and 2,116 ng/mL, as measured in plasma and urine at onset of symptoms, respectively. Acknowledgment of these very high I-FABP levels would perhaps have justified a much earlier surgical intervention.

In many centres clinical deterioration despite maximal conservative therapy is considered an indication for surgical intervention. In our study, there was no difference in urine or plasma I-FABP levels between patients in whom a radiologically proven perforation was the indication for surgery and patients in whom clinical deterioration despite maximal therapy warranted an intervention. However, numbers are limited and further research is needed, as deciding to take a patient to theatre in absence of signs of a perforation forms one of the major challenges for physicians treating children with NEC.

We recognize some limitations of our study. First of all, it was limited by the relative small number of patients. Secondly, NEC in preterm or in full term infants is considered a distinct entity [19]. Since all infants included in our study had a gestational age of less than 36 weeks, we limited our discussion to NEC in preterm patients. Conversely, if indeed NEC in term neonates is a different entity, this might be considered a strong point of our study because it reduced heterogeneity of our subjects. Thirdly, because we used a different ELISA to measure I-FABP than is customary in recently published papers, we refrained from quantitatively comparing our results with those of studies using different ELISAs [6,7,9,15,20]. The lack of standardized I-FABP preparations and/or reference sera limits doing so [12]. We did not use a control group of ‘healthy’ controls. Definition of a control group is extremely difficult in the setting of premature children with different diseases and different gestational ages. In the present study, patients with NEC symptoms who ultimately were diagnosed with another disease were considered the control group, as this reflects daily practice. Currently, performing an ELISA to measure I-FABP levels takes several hours and remains an experimental procedure, but progress is being made to develop a point-of-care test.

## Conclusion

Intestinal fatty acid-binding protein, whether measured in plasma or urine, identifies NEC patients among preterm infants with non-specific Bell’s stage I symptoms. Even more so, in NEC patients I-FABP levels predict complicated disease during the first stages of NEC. By using serial measurements and clearly defined cut-off values, the course of disease can be closely monitored and predicted. Highest I-FABP levels are present during the first 24 hours after symptom onset and followed by gradually diminishing I-FABP levels.

## Supporting Information

S1 Dataset (XLSX)
